# Navigating the Zika panic

**DOI:** 10.12688/f1000research.9370.1

**Published:** 2016-08-04

**Authors:** Nathan D. Grubaugh, Kristian G. Andersen

**Affiliations:** 1 Department of Immunology and Microbial Science, The Scripps Research Institute, La Jolla, CA, 92037, USA; 2 Scripps Translational Science Institute, La Jolla, CA, 92037, USA; 3 Broad Institute of MIT and Harvard, Cambridge, MA, 02142, USA

**Keywords:** Zika virus, arbovirus, virus emergence, microcephaly, antibody-dependent enhancement, Brazil Olympics 2016

## Abstract

The epidemics of Ebola virus in West Africa and Zika virus in America highlight how viruses can explosively emerge into new territories. These epidemics also exposed how unprepared we are to handle infectious disease emergencies. This is also true when we consider hypothesized new clinical features of infection, such as the associations between Zika virus infection and severe neurological disease, including microcephaly and Guillain-Barré syndrome. On the surface, these pathologies appear to be new features of Zika virus infection, however, causal relationships have not yet been established. Decades of limited Zika virus research are making us scramble to determine the true drivers behind the epidemic, often at the expense of over-speculation without credible evidence. Here we review the literature and find no conclusive evidence at this time for significant biological differences between the American Zika virus strains and those circulating elsewhere. Rather, the epidemic scale in the Americas may be facilitated by an abnormally warm climate, dense human and mosquito populations, and previous exposure to other viruses. Severe disease associated with Zika virus may therefore not be a new trait for the virus, rather it may have been overlooked due to previously small outbreaks. Much of the recent panic regarding Zika virus has been about the Olympics in Brazil. We do not find any substantial evidence that the Olympics will result in a significant number of new Zika virus infections (~10 predicted) or that the Olympics will promote further epidemic spread over what is already expected. The Zika virus epidemic in the Americas is a serious situation and decisions based on solid scientific evidence - not hyped media speculations - are required for effective outbreak response.

## Zika’s path from obscurity to severity

Zika virus, a name now synonymous with birth defects by many people, was not always a topic of public health debate. In fact, for 67 years, the virus was mostly ignored (89 publications from 1947 to 2014, compared to 850 over the last 19 months). That is because when Zika virus was discovered in 1947 it was not thought to cause severe enough disease in humans to warrant intense research ^1^. Fast forward to today and people are talking about canceling one of the world’s most watched events, the Olympics, due to the Zika virus epidemic in Brazil ^2, 3^. So, what happened? Did the virus change? Did we misinterpret its threat from the beginning? And will the Olympics this summer really exacerbate the current epidemic or provide new opportunities for Zika virus emergence? Zika virus research is now pouring in fast, but at times at the expense of fast-tracked studies and misinterpretation of results. As a result, there is significant confusion surrounding the Zika virus epidemic and many of the core questions need to be revisited.

It has been suggested that *“The Brazilian strain of Zika virus harms health in ways that science has not observed before”* ^3^ and *“[Africa and Asia] have mostly avoided the post-2013 neurotrophic strains of the virus that are ravaging Brazil”* ^2^. Based on available evidence, however, it is too early to say whether this strain is in fact fundamentally different from other Zika virus strains. Only recently has Zika virus been associated with large outbreaks (since 2007 - Yap Island ^4^) and severe disease such as microcephaly and Guillain-Barr *é* syndrome (since 2013 - French Polynesia ^5^). The epidemic in the Americas has proven to alarmingly increase these trends - 0.5 to 1.5 million suspected infections and ^~^4,000 cases of microcephaly in Brazil alone ^6^. What are the real reasons behind the severity of this epidemic? We will explore aspects of *1*) viral genetics that might alter transmission and pathogenicity in humans, *2*) the ecological conditions in the Americas, *3*) the potential impact of dengue virus on Zika virus-associated pathology, and *4*) how small sample sizes and under reporting may have skewed our previous assumptions of Zika virus and the disease it can cause. Using this knowledge, we will discuss whether a global event like the Olympics would really impact further Zika virus emergence and the expansion of the epidemic.

## Is Zika virus different today than it was when it was first discovered?

Undoubtedly, yes, Zika virus circulating today is genetically different from the Zika virus of the past. A key aspect of Zika virus is that it has an RNA genome. Central features of RNA virus biology is that these viruses replicate, produce large population sizes, but do so with lots of errors (mostly because their polymerases lack proofreading mechanisms, adding ^~^1 mutation per genome replication) ^7– 9^. Therefore, all RNA viruses have the ability to evolve fast relative to most DNA-based organisms ^10^, and Zika virus has evolved into at least two distinct lineages: African and Asian ^11^. The viruses circulating in the Americas belong to the Asian lineage, which, to the best of our knowledge, originated in East Africa ^12^. Comparing the genetics of the first discovered Zika virus strain from 1947 (Uganda, strain MR766) to the strain currently circulating in the Americas (2015 Puerto Rico, strain PRVABC-59) reveals mutations in >1,100 nucleotide positions ( ^~^89% similarity), and confirms that yes, the viral genome is different. While the genetic makeup has changed - as is expected - the more important question, however, is whether this means that the currently circulating strain of Zika virus has a fundamentally different “behavior” ( *i.e.,* phenotype)?

Unfortunately, we are critically lacking comparative studies to directly address whether genetic changes in the virus are significantly contributing to the epidemic. For example, are there differences in mosquito vector competence ( *i.e.,* ability of a population of mosquitoes to transmit the virus) between Zika virus strains in Africa and the Americas? While studies have shown that competence of the suspected Zika virus vectors in the Americas, *Aedes aegypti* and *Ae. albopictus*, can vary between mosquito populations ^13^, the influence of Zika virus genetics has not been tested. There is, however, precedence for mosquito-borne viruses to adapt to local mosquitoes, increasing their epidemic potential. This happened with chikungunya virus during the Indian Ocean epidemic ^14^ and West Nile virus during its invasion of the United States ^15, 16^. Hence there could be some yet-to-be discovered Zika virus mutations that facilitate faster transmission and increased rates of mosquito infection. A lot of work needs to be directed towards lab and field mosquitoes studies to actually determine if this has occurred and whether Zika virus could have adapted to enhance local transmission. At this point, however, there is no evidence that the Zika viruses in the Americas have adapted to the local conditions or can be transmitted any more efficiently than previous strains.

Perhaps even more controversial and urgent are the questions: is the Brazilian strain of Zika virus more capable of *1*) being transmitted during pregnancy or *2*) causing neuropathogenesis leading to complications such as microcephaly ^17^ and Guillain-Barré syndrome ^5^ than strains from Africa or other Asian strains? Several recent laboratory studies have shown that Zika virus can infect placental cells ^18– 20^, be vertically transmitted to offspring during pregnancy ^21, 22^, target and replicate in neuronal cells ^20, 23, 24^, and cause birth defects ^25^ ( *in vivo* in mice, *in vitro* with human cells, organoids, and organ explants). These studies, however, were conducted with a variety of Zika virus strains indicating that some of these phenotypes are common features of the virus, irrespective of the strain. In fact, the virus was first isolated in 1947 by injecting serum from a febrile rhesus macaque directly into a mouse brain ^1^ and a subsequent paper published in 1971 showed that the same Zika virus strain could cause neurological disease in mice ^26^. While these observations may not be that surprising - the mice were infected intracerebrally after all (as is common for these types of experiments) - these early experiments already demonstrated that Zika virus can replicate and cause pathology in neurons. Together, these studies suggest that vertical transmission and neuropathogenesis are not specific attributes of the Brazilian strain and perhaps Zika virus has always been capable of this.

So were those ancestral strains from the 1940’s to 1970’s in Africa reported to cause mild disease actually misunderstood? We know that people in some areas of Africa had high seroprevalence to Zika virus ( *e.g.,* ^~^30% in Nigeria during the early 1970s ^27^). Perhaps disease associated with Zika virus was just overlooked in Africa because of the many other diseases such as malaria, acquired immune deficiency syndrome (AIDS), and tuberculosis that ravage the continent. In the Americas, a large number of Zika virus-naive people ( *i.e.,* without previous immunity) are getting infected at the same time, which may reveal previously unknown clinical features of viral infection. Finally, genetic analysis of Zika virus strains has yet to discover any appreciable patterns associated with adaptation towards humans, vector species, or disease outcome ^28^. This is not to say that it has not occurred, only that at this point in time our sampling is too insufficient to make any conclusions. Therefore, more experimental evidence is required before we can say whether Zika virus genetics or phenotype has changed in any significant way.

## Why is the epidemic in the Americas so bad?

Zika virus is not the first, nor likely the last mosquito-borne virus, to explosively emerge in the Americas. In 1999, West Nile virus was introduced into the New York area and quickly spread across the continent, killing thousands of people and millions of birds (reviewed by 29). Even more recently, in 2013, chikungunya virus emerged throughout the Caribbean and much of the tropical regions in the Americas (reviewed by 30). By 2015, there were already more than one million suspected cases ^31^. Since chikungunya and Zika viruses share similar ecologies (humans and *Ae. aegypti*), the current Zika virus outbreak should not be so surprising, given recent histories. Even the 2007 Yap Island outbreak gave us some indication of its potential - it is estimated that 73% of the population became infected with Zika virus ^4^. A large outbreak in the Americas almost seemed inevitable, but why?

Well, likely because the Americas are home to large and dense populations of hosts (humans) without previous Zika virus immunity, and vectors (mosquitoes) capable of transmission. The climate may also have contributed to the scale and intensity of the epidemic; 2015 was the warmest year on record in the Americas ^32^, which could have enhanced Zika virus transmission. Warmer temperatures can increase mosquito abundance, survival, blood feeding rates, and vector competence ^33– 36^. Therefore, the extreme circumstances caused by *El Niño* and global climate change may have contributed to a higher density of mosquitoes ^37^. Together, these factors represent an ideal recipe for an infectious disease epidemic.

The unfortunate surprise was the discovery of an association between severe neurological complications and Zika virus infection, especially among newborns ^38^. This, however, could just be a consequence of numbers and reporting. Previous outbreaks may have missed these links because they were too small. In Brazil, the current estimate is that between 1–13% of pregnant women who become infected with Zika virus in their first trimester will deliver babies with microcephaly ^17, 39^. That is 8-650× the baseline microcephaly rate of 0.02–0.12% per live birth ^17, 39^. The largest previous Zika outbreak - which occurred in French Polynesia - had an estimated size of 30,000 infections based on serological evidence (11% of the 270,000 people) ^40^. Retrospective analysis of first trimester pregnancies indicated that about 1% of Zika virus infections resulted in babies born with microcephaly - a total of eight cases ^41^. During the Yap Island outbreak, researchers estimated that about 5,000 of the 7,000 inhabitants over the age of three became infected ^4^. Based on Yap State census reports ^42^, roughly 200–300 women may have been pregnant during the outbreak, and only about ⅓ would have been in the first trimester. If the previous estimates were accurate during the Yap Island outbreak, then it may be possible that no babies were born with microcephaly just because there were not enough infected pregnant women for the chance occurrence. The current epidemic in the Americas, including Brazil, may therefore just seem more severe because there are more infected people to detect rare pathological complications such as microcephaly.

Discovering new disease associated with a particular virus only after a large outbreak is not unique to Zika virus. In fact, we can learn from previous epidemics with different viruses, such as West Nile virus. Prior to the 1990’s, West Nile virus was only known to cause sporadic outbreaks with a few cases of severe neurological disease. An outbreak in Romania ^43^, however, redefined our perception of the virus. From 1996–1997, there were more than 500 clinical West Nile cases with a fatality rate approaching 10%, representing the largest mosquito-borne virus outbreak in Europe in more than a decade. Other outbreaks in urban areas soon followed, occurring in Russia ^44^, Israel ^45^, and the United States ^46^, all of which included neurological disease in about 1% of the cases ^29^. While genetic changes to the virus may account for some of the increase in severity ^47^, most of it can be attributed to previously underestimating its severity due to small sample sizes. Many parallels can be made between what happened with West Nile virus and the sudden increase in Zika virus associated disease during its current emergence.

There may also be immunological explanations for pathology associated with Zika virus infection in the Americas. Zika and dengue viruses co-occur in many parts of the world. The fastest growing numbers of dengue cases occur in Latin America and the Caribbean with more than 10 million apparent infections a year ^48^, a ^~^250% increase since 1990 ^49^. One interesting hypothesis is that antibodies produced from a previous dengue virus infection may enhance subsequent Zika virus infection ^50– 53^. The proposed mechanism is that antibodies targeting dengue virus can bind to Zika virus during an active infection, but cannot always neutralize it. Instead, the bound antibodies can actually help Zika virus infect monocytes by attaching to the cell surface receptors (Fc gamma) and mediating efficient entry. This process of antibody-dependent enhancement is also known to occur between different serotypes of dengue virus and is a risk factor for severe dengue disease (reviewed by 54). Since 2010, between 600,000 and 1.6 million annual dengue virus cases in Brazil have been reported ^55^. Therefore the high incidence of dengue virus infection may be increasing the observed pathogenicity of Zika virus in the Americas. On the other hand, dengue and Zika viruses co-occur elsewhere, so the Americas may not be so unique. Indeed, further research is urgently needed to determine if dengue virus is not only exacerbating the Zika virus epidemic in the Americas, but also anywhere the two viruses co-circulate.

## How many visitors will become infected with Zika virus during the Olympics?

Now turning our attention from the biology and genetics of Zika virus, to the different risks associated with Zika virus and the Olympics. There are two main risks to consider: *1*) the risk of further spread and *2*) personal risk to visitors. These are two very different questions, but often they get blurred together. Here we will discuss them separately. First, how many of the expected half a million visitors to Rio de Janeiro during the summer Olympics this August will become infected with Zika virus? Massad *et al.* predict that the numbers of individuals acquiring Zika virus during the Games is low - 1 in ^~^30,000 to 100,000 people ^56^. This translates to only 5 to 15 visitors during the 3-week games will locally acquire Zika virus. The same model was used to predict that 3 to 59 visitors would become infected with dengue virus during the 2014 World Cup ^57^. The actual reported number was three, suggesting that such estimates are relatively accurate ^58^. Another group estimated that 3 to 80 visitors will become infected with Zika virus during the Games ^59^. Based on previous experience and scientific evidence, we might therefore only expect that about 10 people traveling to Brazil for the Games will get infected with Zika virus. Compared to the overall number of cases, that is an astonishingly low number.

One main reason behind the few predicted Zika infections of visitors is that August is winter in Brazil, so mosquito densities will have declined ^60, 61^. That alone will severely decrease the likelihood of exposure to Zika virus through fewer mosquito “bites”. The remaining risk is dependent upon the *1*) mosquito species, *2*) proportion of infected mosquitoes, and *3*) transmission rates upon blood feeding. A recent report helped to validate our assumption that *Ae. aegypti* is vectoring Zika virus in at least some parts of the Americas ^62^, though other species may be involved ^63^. The proportion of *Ae. aegypti* that are infected with Zika virus at any given time, however, is unclear. *Ae. aegypti* infection rates range from extremely low (unpublished data suggesting only a few Zika virus RNA-positive mosquitoes among thousands tested) to very high (5–17% near homes of suspected Zika patients ^62^). If the infection rates are similar to chikungunya or dengue viruses, then we can expect that 1–2% of *Ae. aegypti* are infected with Zika virus ^64, 65^. Moreover, only a small portion (5–50%) of infected mosquitoes can actually transmit the virus ^13^. So even if you get fed upon by hundreds of mosquitoes, odds are that you will not get exposed to Zika virus.

## Will the Olympics enhance the further spread of Zika virus?

The world is interconnected. Zika virus and many other mosquito-borne viruses have already utilized this interconnectivity to travel great distances. Does a global event like the Olympics really enhance this problem? One estimate indicates that 100 to 400 people infected with Zika virus will enter Europe in 2016 due to normal travel from endemic regions ^66^. That is already 7–80× greater than the number of people predicted to become infected during the Games ( ^~^10 - see above). In reality, not enough people will get infected with Zika virus while visiting Brazil for three weeks to have a significant impact on the already expected viral spread. Unfortunately, Zika virus will spread, just as dengue, chikungunya, and West Nile viruses have done before. Holding the Olympic Games in Brazil will have no, or extremely limited, effect on this process.

To really understand the risks, let’s use an example. If a person is infected with Zika virus and returns to their home country, what are the real chances that the infected person could initiate local mosquito-borne transmission? The answer is largely dependent on the local conditions. Specifically, does the environment support enough competent mosquitoes that feed on humans to facilitate transmission? And will such transmission be sustained? Most of Europe, the United States, and other temperate regions cannot support local Zika virus transmission because they either *1*) have an environment that is inhospitable to *Ae. aegypti* (or other susceptible mosquitoes) or *2*) have infrastructure to minimize the risks of mosquito exposure ( *e.g.,* air-conditioned homes and mosquito management programs) ^67^. Much of the tropical and subtropical regions of the world, however, have suitable environmental conditions to support Zika virus transmission ^67^. Spread to many of these places is not concerning, because they already have autochthonous (local) transmission of Zika virus. The Centers for Disease Control and prevention (CDC) recently conducted an assessment of countries that could be at risk of Zika virus importation following the Olympics ^68^. They suggest that Angola and China (among other countries) could be at risk because *1*) they are currently not reporting autochthonous Zika virus transmission, *2*) they likely have conditions to support transmission ( *i.e.,* dense human populations and *Ae. aegypti*), and *3*) there is a high amount of expected air travel from Brazil. In short, it really depends on the home country of the traveler, what season it is, their economic status, whether they can protect themselves from mosquitoes, and many other variables. While a single traveler could be responsible for a new outbreak (as suggested for the Zika virus introduction into Brazil ^28^), in all likelihood these events are extremely rare.

The ‘single introduction’ hypothesis put forward by Faria *et al.* ^28^ has often (wrongly) been used to suggest that it only takes one infected traveler to start an outbreak (i.e., giving the sense that this could happen anytime) ^2^. That is not correct and was also not suggested by the authors. Instead, what the Faria *et al.* data show, is that the chance of starting an outbreak is extremely low. If it had been high, we would have seen multiple introductions of Zika virus into Brazil (and elsewhere), due to travelers arriving from Zika endemic countries. We don’t see that, hence it likely takes many - not just one - infected travelers for the chance occurrence to start an outbreak.

Direct human-to-human transmission is another possible route of Zika virus infection. These routes notably include transmission from mother to child during pregnancy and sexual transmission from a man to a woman ^69^. Other forms of human-to-human transmission scenarios also appear to exist ^70^. Therefore, could sustained Zika virus transmission occur without mosquitoes and should this be a concern for further spread of the epidemic? Again, let’s use an example: an infected man returns home from the Games and has sex with his partner. There are numerous reports of Zika virus infection associated with sex with a man (or woman ^71^) returning from an endemic region ^72– 74^. Therefore, in this scenario, there is immediate risk to his partner. Importantly, however, in each of these reports, Zika virus spread was limited to just those single contacts. Thus, sex and other modes of direct contact with an infectious individual is highly unlikely to lead to sustained transmission in a new population. It has also been estimated that the role of sexual transmission in Brazil is minimal compared to mosquitoes ^75^, and without mosquitoes, transmission would dissipate. The single most compelling piece of evidence to support that Zika virus is primarily mosquito-borne is that it is only known to occur in regions with *Ae. aegypti* ^67^. Therefore, Zika virus is still considered to be primarily transmitted by mosquitoes and sexual transmission (or other yet-to-be-discovered human-to-human means of transmission) will likely not expand the expected range of the Zika virus epidemic.

## Take-home message

Our rapidly expanding knowledge about Zika virus is starting to reveal important information about the current epidemic and suggests that we may have misjudged its epidemic potential for decades. We explored four key areas to demonstrate how the epidemic severity may be more related to the conditions in the Americas rather than new disease caused by Zika virus. *1*) There is currently no definitive evidence that the strain of Zika virus in Brazil has altered potential for transmission or pathogenicity in humans compared to the strains circulating in Africa and Asia (although this does not mean that the Brazilian strain does not have an altered phenotype compared to other strains, only that no good evidence is currently available to suggest that is the case). *2*) Major factors for the scope of the epidemic were likely large urban settings housing people without immunity and an abnormally warm climate leading to a large population of mosquitoes. *3*) Previous exposure to dengue virus could increase Zika virus disease severity, though such a connection is yet to be demonstrated as an important risk factor. *4*) The recent associations of some Zika virus infections with severe neurological conditions, such as microcephaly and Guillain-Barré syndrome, may be simply a reflection of sample sizes - large numbers of infections are often required to discover rare pathologies.

The risks regarding Zika virus and the Olympic Games in Brazil are *1*) whether it will enhance the epidemic spread and *2*) personally to people attending the games. The numbers of Olympic visitors expected to get infected with Zika virus in Brazil and travel home is far lower than the total numbers of these occurrences already expected to happen throughout the year. Therefore, the Olympics will not be a significant conduit for further epidemic spread. There is a personal risk of infection, though it is also predicted to be low. Obviously, pregnant women have the greatest risk as they could pass the virus to their developing fetus, with the possibility of causing severe neurological complications. Therefore each family needs to evaluate the consequences and likelihood of Zika virus infection to determine if they should travel to any region of the world with active Zika virus transmission. The Zika virus epidemic is a severe problem, but decisions should be based on scientific evidence ^78, 79^ and not fear-mongering ^2^. These should be lessons to keep in mind when we argue about some other relatively unknown virus before the start of Tokyo Olympics in 2020.
