# Supplementary material for: Navigating the Zika panic
Source: F1000Res. 2016 Aug 4;5:1914. [Version 1] doi: 10.12688/f1000research.9370.1 (PMC5040150; doi:10.12688/f1000research.9370.1)
Supplement: Peer review history

# Peer review: Referee response for version 1

**DOI:** 10.5256/f1000research.10091.r16130

**Published:** 2016-09-23

**Authors:** Gonzalo Moratorio, Bryan.C Mounce

**Affiliations:** 1 Vignuzzi Lab (Viral Populations and Pathogenesis Unit), Pasteur Institute, Paris, France

In this interesting and well-executed review, Grubauch and Andersen 'navigate' the literature to provide a clean, well-driven and totally enjoyable text about the current situation generated for the Zika outbreak in the Americas. Based on the bibliography, they conclude there is no scientific evidence that the Brazilian Zika strain presents a higher pathogenicity compared to others circulating elsewhere. In addition, as was discussed in previous reports, they agreed with the role played by urban and weather factors to particularly enhance this outbreak.  The authors also believe that is totally necessary to confirm the connection between Dengue and Zika as a risk factor in disease severity. Finally, given the sample size of the recent outbreak, we could have under estimated rare diseases associated to this virus in the past.

  The article itself is very nicely written and provides a very balanced viewpoint of Zika virus, which is something that has been lacking from several media sources. The authors have put significant thought into the attributes that affect the severity of the outbreak, but perhaps the most important statements concerning the evolution of the virus and the neurological phenotypes recently observed. With their calculations for the number of microcephaly cases that could have been detected on Yap Island (quite possibly none due to the size of the outbreak), they suggest that statistics and surveillance, rather than genetic differences, affect disease severity. Of course, additional research is necessary into understanding the severity of the Zika virus outbreak and its connections to microcephaly and Guillain-Barre syndrome and whether the genetic differences between the strains of Zika virus are or are not responsible for these newly observed phenotypes.

 The authors also expand on the details on how Zika virus could infect a traveler in Brazil and then induce an outbreak in their home country, which they estimate to have a much lower probability than popular media sources might suggest. Though the commentary surrounding the possibility of the Olympics enhancing Zika virus comes after the games, the thoughtful consideration of the risks supplies rational thinking that has been lacking, especially when juxtaposed with the hysteria prior to the games. The review superbly quells the hyperbole that has surrounded the Zika outbreak in the Americas. Regardless of this low possibility of traveler-associated transmission of Zika virus in new locales, regions with the proper conditions for Zika virus transmission should remain vigilant, and continued campaigns of mosquito control, especially given the breadth of viruses spread by mosquitoes (including dengue, chikungunya and West Nile viruses).

 We would like to highlight that the authors didn’t cite any of their previous works, which surprised us in a positive way, showing the author’s will to deliver a clean and not biased opinion about the topic.

 Minor comments:

 The article is written for any kind of reader, which we found excellent, but in that case would be nice to clarify what is *El Niño*. Even if wouldn’t be necessary for most of the readers.  

 The paragraph that starts with “One main reason behind …August is winter in Brazil…”

 Brazil is huge, is the he largest country in South America and in the Southern Hemisphere. Is placed 5 ^th^ in the list sovereign states and dependencies by area in the world. Particularly, the weather in Rio, still in august, can be warm enough for mosquitos. Last August the lower temperature registered was 71 F and the higher 78 F.

We have read this submission. We believe that we have an appropriate level of expertise to confirm that it is of an acceptable scientific standard.

# Peer review: Referee response for version 1

**DOI:** 10.5256/f1000research.10091.r16240

**Published:** 2016-09-12

**Authors:** Eduardo Massad, Claudio J Struchiner

**Affiliations:** 1 School of Medicine, University of São Paulo, São Paulo, Brazil; 2 London School of Hygiene & Tropical Medicine, London, UK; 3 Program of Scientific Computing, Fundação Oswaldo Cruz (PROCC, FIOCRUZ), Rio de Janeiro, Brazil

In an attempt to justify the disastrous Iraq invasion in 2002, the then 13th Secretary of Defense of the USA, Donald Rumsfeld, stunned reporters when he uttered his (in)famous reflection on "known knowns",  "known unknowns" and "unknown unknowns" (Rumsfeld, 2011).

 In their interesting review on Zika epidemics, Grubauch and Andersen address the panic ensued by the overwhelmed number of cases in Latin America and elsewhere. They call the attention to several "known knowns" and "known unknowns" of the current Zika epidemics, although they do not shy away of pointing to possible "unknown unknowns".

 Grubauch and Andersen find no conclusive evidence in the literature for significant biological differences between the Latin American Zika virus strain and those circulating elsewhere. They argue that the epidemic scale in the Americas has been influenced by climate, humans and mosquitoes population densities and local prevalence of other viruses, in particular flaviviruses, like dengue and yellow fever. The severe neurological abnormalities associated with Zika virus would not be a new trait of the Latin America strain, but rather may have been overlooked due to previously small outbreaks.

 Every time the world is hit by an emergent or re-emergent pathogen with pandemic potential, panic ensues (Amaku et al., 2016). This is understandable due to previous history of recurrent pandemics, like the medieval Black Death or the 1918 Spanish Flu, which killed millions of people in Europe and around the world (Massad et al., 2007; Burattini, Coutinho & Massad, 2009). After the Pasteur-Koch germ theory was proposed, the previously "unknown unknowns"  could explain the panic. The recent panic observed when SARS, H5N1 and more recently MERS-CoV emergence, or the current Zika, should not be justified due to the prompt discovery of the ultimate cause and the possible control mechanisms. Unlike Londoners of the XVII century (Bell, 2001), who burnt witches and culled thousands of cats (by the way, both excellent rat killers), we have the scientific tools and mechanisms to face the threat with the necessary rationality.

 In the case of Zika virus, however, many "known unknowns" still remain, like the true phenotypic repercussion of genotypic differences between the strains circulating in different parts of the world, differences in mosquito competences, the actual number of people that has already been infected, the competition by the vector of different viruses transmitted by the same aedes mosquitoes, vertical transmission of the virus in hosts and vectors, neuropathogenic potential of different strains, cross-immunity, antibody-enhancement by previous infection, just to mention a few. In addition, it is not well known the actual role of sexual transmission of Zika in triggering or maintaining an outbreak. As for the "unknown unknowns", just time will disclose.

 One important unknown currently being discussed in the literature is whether Zika will disappear from the affected regions and whether and when it will recur.  Mathematical models, very useful in the understanding and control of previous epidemics, have been widely applied in an attempt to describe and make prediction about the current Zika outbreak (Massad et al., 2016)). We now know that the Basic Reproduction Number, *R*_0_, of Zika is around 3, slightly higher than that of Dengue (Coelho et al., 2008) and it is even possible to predict the likelihood of Zika being exported to unaffected regions of the world, either causing a single and self limited outbreak or establishing itself, depending on whether localis lesser or greater than 1.

 For the sake of completeness, two additional speculative unknowns are worth mentioning. The circulation of Zika virus and its potential interaction with dengue raises new concerns regarding vaccination strategies against the latter. The subtle trade-off justifying the recommendation of the vaccine might no longer hold. On the other hand, the patchy distribution of serious outcomes due to Zika has not been satisfactorily explained. Our navigation map cannot overlook the apparent clustering of cases of microcephaly reported in northeastern Brazilian states so far.

 We think that the many "known unknowns" related to Zika epidemic explored in this paper are worthwhile being published and we are sure that Grubauch and Andersen reflections could help bring science and rationality to the fore, soothing the perhaps unjustifiable panic. Althoug Grubauch and Anderson do not cross all the t's nor dot all the i's, they at least show us all the uncrossed t's and undotted i's of the current Zika "unknowns" epidemic. This definitively qualify their paper for publication.

 Finally, for the record: the World Health Organization officially declared - zero cases of Zika during the Olympic Games!

We have read this submission. We believe that we have an appropriate level of expertise to confirm that it is of an acceptable scientific standard.

## References

[1] AmakuMBurattiniMNCoutinhoFALopezLFMesquitaFNaveiraMCPereiraGFSantosMÉMassadE: Estimating the Size of the HCV Infection Prevalence: A Modeling Approach Using the Incidence of Cases Reported to an Official Notification System. *Bull Math Biol*.2016;78(5) : 10.1007/s11538-016-0170-4 970-90 10.1007/s11538-016-0170-4 27160282

[2] BellNG: The Great Plague in London. *The Folio Society*.2001;

[3] BurattiniMNCoutinhoFAMassadE: A hypothesis for explaining single outbreaks (like the Black Death in European cities) of vector-borne infections. *Med Hypotheses*.2009;73(1) : 10.1016/j.mehy.2009.01.036 110-4 10.1016/j.mehy.2009.01.036 19264416

[4] CoelhoGEBurattiniMNTeixeiraMda GCoutinhoFAMassadE: Dynamics of the 2006/2007 dengue outbreak in Brazil. *Mem Inst Oswaldo Cruz*.2008;103(6) :535-9 1894932110.1590/s0074-02762008000600004

[5] MassadEBurattiniMNCoutinhoFALopezLF: The 1918 influenza A epidemic in the city of São Paulo, Brazil. *Med Hypotheses*.2007;68(2) : 10.1016/j.mehy.2006.07.041 442-5 10.1016/j.mehy.2006.07.041 17011139

[6] MassadETanSHKhanKWilder-SmithA: Estimated Zika virus importations to Europe by travellers from Brazil. *Glob Health Action*.2016;9:31669 2719326610.3402/gha.v9.31669PMC4871896

[7] RumsfeldD: Known and Unknown: A Memoir. *Penguin Books*.2011;

# Response

**Published:** 2016-09-12

**Authors:** Nathan Grubaugh

**Affiliations:** Scripps Research Institute, La Jolla, USA

Fantastic review! Thank you for taking the time and pointing out some of the issues that we did not get into, mainly because we were already >4000 words! There are so many unknowns of many varieties, we just ask for over-speculation to be tempered until the science can catch up.

 No new Zika virus infections during the Olympics is amazing news!
